# Supplementary material for: Periosteal skeletal stem cells can migrate into the bone marrow and support hematopoiesis after injury
Source: eLife. 2025 May 22;13:RP101714. doi: 10.7554/eLife.101714 (PMC12097789; doi:10.7554/eLife.101714)
Supplement: Supplementary file 1. [file elife-101714-supp1.docx]

**Supplementary Table 1: Flow Cytometry Antibodies**

| **Antibody** | **Clone** | **Fluorochrome (Manufacturer)** |
| --- | --- | --- |
| anti-CD45 | 30-F11 | APC-Cy7 (Invitrogen), Pacific Blue (Biolegend) |
| anti-Ter119 | Ter-119 | APC-Cy7 (Invitrogen), Pacific Blue (Biolegend) |
| anti-CD31 | 390 | PE-Cy7 (Biolegend), AF647 (Biolegend) |
| anti-CD51 | RMV-7 | biotin (Biolegend), PE (Invitrogen) |
| anti-CD200 | OX-90 | APC (Biolegend) |
| anti-CD140α | APA5 | PE-Cy7 (Invitrogen), APC (Biolegend) |
| anti-strepavidin | (n/a) | FITC (Invitrogen), APC-Cy7 (Invitrogen) |
| anti-CD11b | M1/70 | AF647 (Biolegend) |
| anti-CD4 | GK1.5 | PE-Cy7 (Invitrogen) |
| anti-CD8a | 53-6.7 | PE-Cy7 (Invitrogen) |
| anti-B220 | RA3-6B2 | PerCP-Cy5.5 (Invitrogen) |
| anti-CD45.1 | A20 | PE (Biolegend) |
| anti-CD45.2 | 104 | FITC (Biolegend) |
| anti-Ki67 | SolA15 | FITC (Invitrogen) |
| anti-CD144 | BV13 | AF647 (Biolegend) |
| anti-Gr-1 | RB6-8C5 | APC-Cy7 (Invitrogen) |
