# Supplementary material for: Periosteal skeletal stem cells can migrate into the bone marrow and support hematopoiesis after injury
Source: eLife. 2025 May 22;13:RP101714. doi: 10.7554/eLife.101714 (PMC12097789; doi:10.7554/eLife.101714)
Supplement: Supplementary file 2. [file elife-101714-supp2.docx]

| **Primer** | **Forward Sequence (5’ 🡪 3’)** | **Reverse Sequence (5’ 🡪 3’)** |
| --- | --- | --- |
| *Cxcl12* | 5'-CGCCAAGGTCGTCGCCG-3' | 5'-TTGGCTCTGGCGATGTGGC-3' |
| *Kitl* | 5'-CCCTGAAGACTCGGGCCTA-3' | 5'-CAATTACAAGCGAAATGAGAGCC-3' |
| *Opn* | 5'-AGCAAGAAACTCTTCCAAGCAA-3' | 5'-GTGAGATTCGTCAGATTCATCCG-3' |
| *Angpt1* | 5'-CTCGTCAGACATTCATCATCCAG-3' | 5'-CACCTTCTTTAGTGCAAAGGCT-3' |
| *Vcam1* | 5'-GACCTGTTCCAGCGAGGGTCTA-3' | 5'-CTTCCATCCTCATAGCAATTAAGGTG-3' |
| *Cdkn1a* | 5' CCTGGTGATGTCCGACCTG-3' | 5' CCATGAGCGCATCGCAATC-3' |
| *Cdkn1c* | 5-CGAGGAGCAGGACGAGAATC-3' | 5' GAAGAAGTCGTTCGCATTGGC-3' |
| *Cdk4* | 5'-ATGGCTGCCACTCGATATGAA 3' | 5' TCCTCCATTAGGAACTCTCACAC-3' |
| *Sod1* | 5'-TGGTGGTCCATGAGAAACAA-3' | 5'-GTTTACTGCGCAATCCCAAT-3' |
| *Gls* | 5' TTCGCCCTCGGAGATCCTAC-3' | 5' CCAAGCTAGGTAACAGACCCT-3’ |
| *Gpx1* | 5'-AGTCCACCGTGTATGCCTTCT-3' | 5'- GAGACGCGACATTCTCAATGA-3'-3' |
| *Postn* | 5'-CCTGCCCTTATATGCTCTGCT-3' | 5'-AAACATGGTCAATAGGCATCACT-3' |
| *Gapdh* | 5'-TGTGTCCGTCGTGGATCTGA-3' | 5'-CCTGCTTCACCACCTTCTTGA-3' |
| *Actb* | 5' GCTTCTTTGCAGCTCCTTCGT-3' | 5' ATCGTCATCCATGCCGAACT-3' |

**Supplementary Table 2: RT-PCR primer sequences**
